# Thermal and Mechanical Characterization of Carbides for High Temperature Nuclear Applications

**DOI:** 10.3390/ma14102689

**Published:** 2021-05-20

**Authors:** Mattia Manzolaro, Stefano Corradetti, Michele Ballan, Riccardo Salomoni, Alberto Andrighetto, Giovanni Meneghetti

**Affiliations:** 1 National Institute of Nuclear Physics—Legnaro National Laboratories (INFN-LNL), Viale dell’Università 2, 35020 Legnaro, Padova, Italy; stefano.corradetti@lnl.infn.it (S.C.); michele.ballan@lnl.infn.it (M.B.); alberto.andrighetto@lnl.infn.it (A.A.); 2 Department of Industrial Engineering, University of Padova, Via Venezia 1, 35131 Padova, Italy; riccardo.salomoni@studenti.unipd.it (R.S.); giovanni.meneghetti@unipd.it (G.M.)

**Keywords:** high-power beam, high temperature, temperature gradient, thermal characterization, mechanical characterization, carbide

## Abstract

In the facilities for the production of Radioactive Ion Beams (RIBs) according to the Isotope Separation On-Line (ISOL) technique, a production target is typically impinged by a high-power primary beam, generating radioactive isotopes for basic research and technological applications. With the aim to guarantee an efficient extraction of the aforementioned isotopes, the production target must work in a high vacuum environment, at temperatures that are usually between 1600 °C and 2200 °C. Its main components are often characterized by intense temperature gradients and consequently by severe thermal stresses. Carbides are widely used for target manufacturing, and in this work a specific method for their thermal and mechanical characterization is presented and discussed. It is based on the comparison between experimental measurements and numerical simulations, with the introduction of the novel Virtual Thermoelastic Parameters approach for the structural verification procedure. High-performance silicon carbides (SiC) are taken as a reference to describe the method. Measured emissivity and thermal conductivity data are presented and discussed, together with the experimental estimation of material limitations for both temperature and stress fields. The aforementioned results can be promptly used for the design process of high-power ISOL targets.

## 1. Introduction

Over the last decades, the scientific community has seen a growing interest in the production of Radioactive Ion Beams (RIBs) according to the Isotope Separation On-Line (ISOL) technique. A key role has been played by important international facilities such as ISOLDE [1], ISAC [2], HRIBF [3], SPIRAL [4] and ALTO [5]. Nowadays, several new facilities are under construction around the world, and the Selective Production of Exotic Species (SPES) project represents the contribution of the Italian National Institute for Nuclear Physics (INFN). The aim is to construct at Legnaro National Laboratories (LNL) an ISOL facility to produce neutron-rich nuclei in the 80–160 amu mass range. They will be generated by means of a 40 MeV, 200 µA primary proton beam, directly impinging on a series of thin uranium carbide discs (composing the production target) that are axially spaced in order to dissipate by thermal radiation the considerable amount of power deposited by protons [6,7,8]. As a rule of thumb, the fission of ^235,238^U, ^232^Th and other long lived actinides is adopted to produce neutron-rich nuclei in a wide mass range. These elements are typically used in their carbide forms in order to allow the target operation at high temperature (typically between 1600 °C and 2200 °C) in high vacuum [9]. Indeed, high temperature resistance, together with other material characteristics such as open porosity, is fundamental to guarantee an efficient extraction of radioactive isotopes from the target material [10,11,12].

For some specific applications, focused on narrow mass ranges, it is possible to use different carbides. An example is silicon carbide (SiC), which is usually irradiated with energetic protons to deliver radioactive Al isotopes (mainly ^26^Al, ^28^Al and ^29^Al). This is the case of the SPES facility, which during the commissioning phase will be operated making use of a SiC target. In this way, radioprotection concerns will be extremely limited with respect to uranium carbide. It will be also possible to take as a reference the SPES SiC target tests carried out at the Oak Ridge National Laboratories (ORNL) in similar conditions [13].

It is important to underline the extremely high-power density characterizing the main SPES target components. Indeed, unlike the current ISOL facilities, which are characterized by much higher primary beam energies [1] (with the beam passing through the target and depositing only a fraction of its power), the 40 MeV proton beam available at the SPES facility will be completely stopped into the target, causing the dissipation of the whole beam power within the small target volume. In this context, an intense research and development activity has started with the aim to study the thermal–structural behavior of target materials. As a first step, all efforts were addressed to the commercial high-performance silicon carbides (SiC) that will be used at the SPES facility during the commissioning phase. In this work, a specific approach for their thermal and mechanical characterization is presented and discussed. The experimental apparatuses and techniques described in [14,15] were taken as a reference, and a novel strategy to identify a stress limit value for the structural design phase was introduced. In the future, the same methodology will be extended to new high-performance materials for ISOL applications [10,11,12].

## 2. Target Design Process and Thermo-Mechanical Characterization of Materials

**Figure 1:**
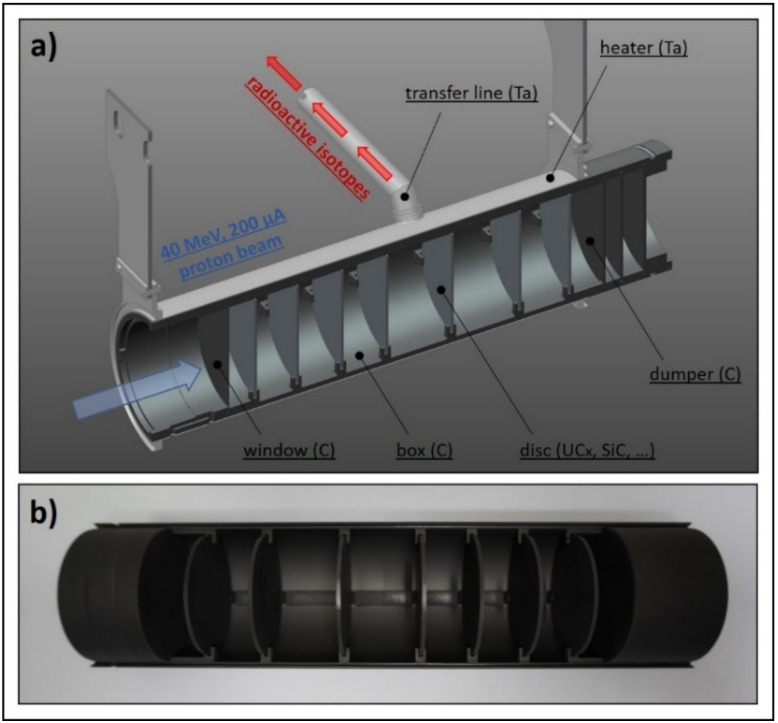
The SPES target architecture; (**a**) CAD view with indication of the main components; (**b**) picture of the open box.

As mentioned above, the SPES production target (see Figure 1) is specifically designed for high-power depositions [6,7] and optimized for the maximization of radiative heat transfer [16]. It is composed of seven co-axial discs made of uranium carbide or other carbides, according to the specific RIB requests. The discs are properly spaced in the axial direction in order to dissipate by thermal radiation the large amount of power deposited by the proton beam. They are characterized by a diameter and a thickness of 40 and 0.8 mm, respectively. Two thin graphite windows located at the frontal part of the target prevent the undesired loss of isotopes, while three graphite dumpers completely stop the proton beam at the rear side. All discs, windows and dumpers are enclosed in a tubular hollow box made of graphite, defining the main target assembly. A tantalum tubular heater is used to heat the target by Joule effect and to control accurately and gradually its temperature during the start-up and the shut-down procedures [17], i.e., when the proton beam power is not stabilized yet. In Figure 1, it is also possible to observe the transfer line. It is a small tube made of tantalum used to transport the radioactive isotopes from the target to the ion source, where they can be ionized and subsequently accelerated by an extraction voltage of approximately 40 kV.

**Figure 2:**
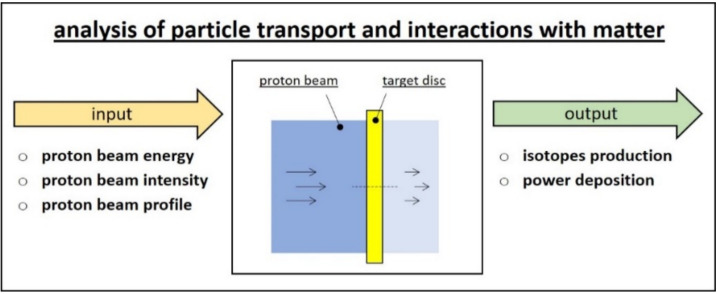
Schematic representation of the analysis of particle transport and interactions with matter.

The design process of high-power targets is complicated and should always include the three following steps:

(a) Analysis of particle interactions with matter;
(b) Thermal analysis;
(c) Structural analysis.

(Step a) is schematically represented in Figure 2. In order to have an accurate estimation of the radioactive isotopes produced by protons’ interaction with the target material, dedicated Monte Carlo codes such as FLUKA and MCNP are adopted [6]. The same codes also provide detailed power deposition maps for the target components directly impinged by the beam (discs, windows, dumpers, box). Of course, the quality and reliability of results are deeply affected by input parameters, in particular by the proton beam energy, intensity and profile.

**Figure 3:**
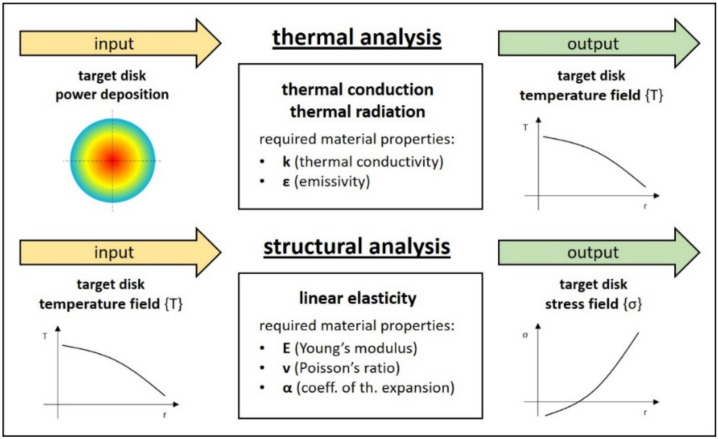
Thermal and structural analyses: inputs, outputs and material properties.

Being the main thermal load for the thermal analysis, power deposition maps are particularly important and directly affect the target temperature field {T}. Accordingly, {T} and temperature gradients constitute the main load for the structural analysis, whose aim is to estimate the stress field {σ} for the target discs (which are surely the most stressed and precious components of the whole target assembly). Both thermal and structural analyses (step b) and (step c), respectively) are schematically represented in Figure 3. They are usually performed with an integrated approach, making use of general-purpose Finite Elements (FE) codes such as ANSYS^®^.

In the case of thermal analyses (step b), steady-state conditions can be considered as a good approximation for typical RIB production phases. In addition, since exclusively thermal conduction and thermal radiation should be included as heat transfer modes (the target works in high vacuum, so thermal convection is not considered), the two material properties required in the pre-processing phase are the thermal conductivity k (materials are considered isotropic) and the total hemispherical emissivity ε (gray-diffuse surfaces are commonly assumed for this kind of radiative heat transfer calculation) [17,18].

Regarding the directly related steady-state structural analyses (step c), the hypotheses of isotropic and linear elastic materials were taken into consideration for the sake of simplicity. Such calculations are clearly used to pass from {T} to the stress field {σ} once the thermoelastic material properties are introduced in the pre-processing phase, which are the Young’s modulus E, the Poisson’s ratio ν and the coefficient of thermal expansion α.

In the final part of the design process, it is fundamental to verify that both the maximum temperature and the maximum stress are well below the material limits, which in this work are indicated as T_LIMIT_ and σ_LIMIT_, respectively. If these conditions are verified, the target design can be approved together with the proton beam working parameters. On the other hand, if T_LIMIT_ and/or σ_LIMIT_ are exceeded, the designer has the possibility to fix the problem in many different ways. Firstly, it is preferable to adjust the proton beam energy, profile and intensity, but this can be performed within certain limits since the RIB production rate must not be affected substantially. Secondly, the target design can be modified in terms of geometry and material selection, but keeping into consideration the constraints related to the surrounding components and the requested nuclear reactions, respectively.

The design approach described above can be actuated only if thermal and structural properties (k, ε, E, ν, α) and limits (T_LIMIT_, σ_LIMIT_) are well known for all the materials constituting the target. Usually, all the components surrounding the target discs (windows, dumpers, heater, …) are made of high-performance commercial materials (graphite, tantalum, …) whose properties are available for a wide temperature range. On the other hand, target discs are manufactured by means of specific home-made procedures. In this case, materials require an accurate thermal and mechanical characterization.

Thermal conductivity (k), total hemispherical emissivity (ε) and temperature limit (T_LIMIT_) can be estimated making use of experimental techniques developed at LNL in the context of the SPES project [14,15]. On the contrary, thermoelastic properties E, ν and α are usually difficult to obtain, especially if temperature-dependent values are required. Sometimes it is possible to assume some values based on the literature, but most of the time these properties are completely unknown. For this specific case, the strategy proposed in this paper is to consider a set of Virtual Thermoelastic Parameters (VTPs). They will be used both for the calculation of {σ} and for the numerical estimation of σ_LIMIT_ (taking as a reference dedicated destructive tests at high temperature). In this way, {σ} and σ_LIMIT_ can be directly compared, with the possibility to evaluate the structural design of the target_._ More details will be described in the following paragraphs.

## 3. Thermal Characterization and Related Experimental Procedures

The aforementioned design process for high-power targets requires the possibility to perform accurate and reliable thermal analyses. As discussed in the previous paragraph, the temperature field in steady-state conditions depends exclusively on k and ε. These two properties are usually unknown for the home-made materials constituting the target discs. With this in mind, a concise description of the methodologies developed at LNL for the estimation of both ε [14] and k [15] is proposed in the following. The high temperature furnace adopted to define T_LIMIT_ is also presented as discussed. Finally, k, ε and T_LIMIT_ values measured for the Hexoloy^®^ silicon carbides SA and SP are reported in the next paragraphs, together with grain size, density, pore size and surface roughness data for both SiC grades. These properties are particularly useful to comment on results, and being referred to commercial materials, they are all available from datasheets, with the exception of surface roughness, which was accurately measured by making use of the mechanical stylus method (Mitutoyo Surftest SJ-210, stylus tip radius R2 µm, detector measuring force 0.75 mN).

### 3.1. Emissivity

**Figure 4:**
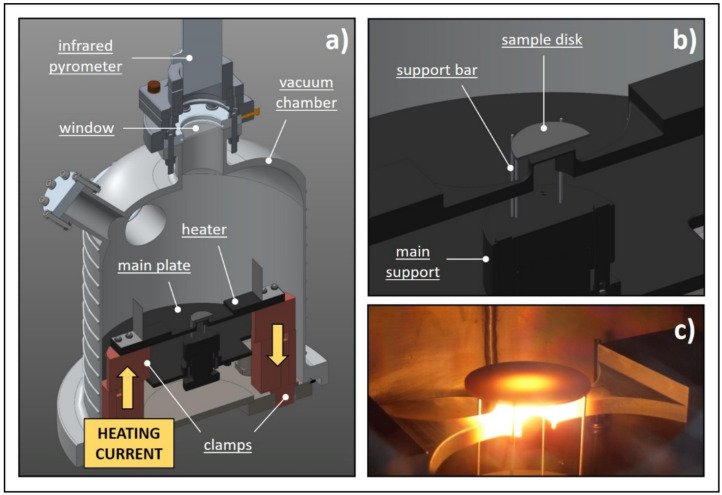
The experimental apparatus adopted for both emissivity and thermal conductivity estimations; (**a**) general CAD view of the whole apparatus; (**b**) detailed CAD view of the sample area; (**c**) picture of the sample area at high temperature.

The experimental set-up used to measure the total hemispherical emissivity ε was entirely designed and constructed at LNL. Its main details are illustrated in Figure 4. A dedicated power supply (I_MAX_ = 1000 A, V_MAX_ = 10 V) directly heats by Joule effect the graphite heater, which is connected to water-cooled copper clamps at its extremities. The heater is designed in order to produce on its top circular surface (diameter equal to 18 mm) a homogeneous temperature distribution at temperature levels up to 2200 °C. Four tungsten bars suspend the sample disc coaxially with respect to the round hot surface of the heater. Sample diameters can range from 30 to 40 mm (the thickness is usually between 0.5 and 3 mm), and the bars (inserted in the graphite main support represented in Figure 4) allow for a precise spacing between the sample disc and the hot surface of the heater, with the possibility to make fine regulations between 0.2 and 5 mm. The spacing is an extremely important parameter that deeply affects both the maximum disc temperature (in the center) and the severity of the gradients (in the radial direction). The temperature profile induced in the sample disc indeed reproduces very closely the temperature field {T} related to the proton beam power deposition, characterized by strong radial temperature gradients. As explained in the following paragraphs, these gradients are particularly important for the study of both k and σ_LIMIT_. For the moment, we simply highlight that such an apparatus allows for fine temperature regulations in the center of the disc (see Figure 4c) by setting up the heater–disc spacing and the heating current coming from the power supply.

All the components described above are closed inside a water-cooled vacuum chamber. Vacuum is kept at approximately 10^−6^ mbar by a rotary pump and a turbomolecular pump placed in series. A high-temperature infrared pyrometer is placed on the top of the vacuum chamber, in proximity of a boro-silicate glass window, which is almost completely transparent to infrared radiation. As discussed in [14], this instrument can operate both in two- and single-color modes. For the materials tested in this work (Hexoloy^®^ silicon carbides SA and SP), the gray-body hypothesis is assumed and temperature in the center of the disc is measured in the two-color mode as specified in the instrument guide and in [14]. At this point, emissivity can be defined using the pyrometer in the monochromatic mode and changing the instrument emissivity value until the measured temperature in the monochromatic mode matches the temperature measured in the two-color mode. Assuming that the measurement direction is normal to the pointed surface, it is fundamental to underline that the emissivity measured in this way is the spectral normal emissivity ε_λ,n_ related to the working wavelength of the instrument in the monochromatic mode (λ = 1.05 μm). If the diffuse gray surface hypothesis (widely used for radiation exchange engineering calculations) is adopted [14], the total hemispherical emissivity ε can be directly approximated with ε_λ,n_. It is important to underline that all data are collected automatically by making use of a computer connected to the instrumentation by a Programmable Logic Controller (PLC).

### 3.2. Thermal Conductivity

The thermal conductivity k of the sample material can be estimated by means of the steady-state method reported in [15] and the experimental apparatus described in the previous paragraph (see Figure 4). At the basis of the method there is a consolidated electrical–thermal finite element model of the experimental apparatus that is capable of simulating both the conduction/radiation thermal problem (convection is not considered in high vacuum) and the electrical one. In steady-state conditions, ε and k must be known for all materials since these are the two properties required to solve the thermal problem. Moreover, since high DC currents pass through the heater and the clamps, the electrical resistivity ρ should be introduced for both graphite and copper to allow the implementation of the electrical problem and the Joule heating effect.

At this point, it is important to highlight that all the aforementioned material properties (ε, k and ρ) were accurately verified during the years through dedicated experimental tests and accurate literature searches [15]. Of course, the only exception is constituted by the sample material (involved exclusively in the thermal problem), for which ε and k are unknown. Now, temperature-dependent emissivity values can be surely estimated using the above described procedure, whereas k is assumed to be temperature dependent through the following quadratic expression:

k = C_0_ + C_1_·T + C_2_·T^2^ (1)

From the observation of Equation (1), it is evident that k is expressed by means of three unknown parameters (C_0_, C_1_ and C_2_) that can be easily represented in vector form as proposed in the following:

**f** = {f_1_, f_2_, f_3_} = {C_0_, C_1_, C_2_} (2)

In this shape, the problem can be regarded as a typical optimization problem, whose goal is the consistency between computed and measured temperatures. The quantification of such consistency is represented by the following residual function:

(3) $$J\left(f\right)=\overset{N_{\mathrm{CS}}}{\underset{i=1}{\sum }}\;{\left[T_{C\_\mathrm{COMP}\_i}\left(f\right)-T_{C\_\mathrm{MEAS}\_i}\right]}^{2}+{\left[T_{P\_\mathrm{COMP}\_i}\left(f\right)-T_{P\_\mathrm{MEAS}\_i}\right]}^{2}$$

where N_CS_ is the number of current steps used to power the heater and so to heat the sample, T_C_COMP_i_ and T_P_COMP_i_ are the computed temperatures at the center and at the periphery of the sample disc, respectively, and T_C_MEAS_i_ and T_P_MEAS_i_ are the correspondent measured values. A specific steady-state temperature field is associated with every current step, for both the numerical model (T_C_COMP_i_, T_P_COMP_i_) and the experimental tests (T_C_MEAS_i_, T_P_MEAS_i_). The optimization process, so the minimization of J with respect to **f**, is performed numerically, making use of the optimization tools implemented in the ANSYS^®^ environment, allowing the determination of the quadratic expression for k.

### 3.3. Temperature Limit

As mentioned in the previous paragraphs, the proton beam working parameters must be accurately set in order to guarantee that the maximum temperature of the target discs stays well below the temperature limit (T_LIMIT_) of their material, which is usually between 1600 °C and 2200 °C.

In our case, T_LIMIT_ is estimated as the highest temperature level for which the observed mass loss (due to sublimation in high vacuum) stays below 0.5%. Numerous tests were performed, keeping the sample at a constant reference temperature for 24 h (at similar vacuum levels with respect to typical ISOL target working conditions) and measuring its weight just before and after every single test.

**Figure 5:**
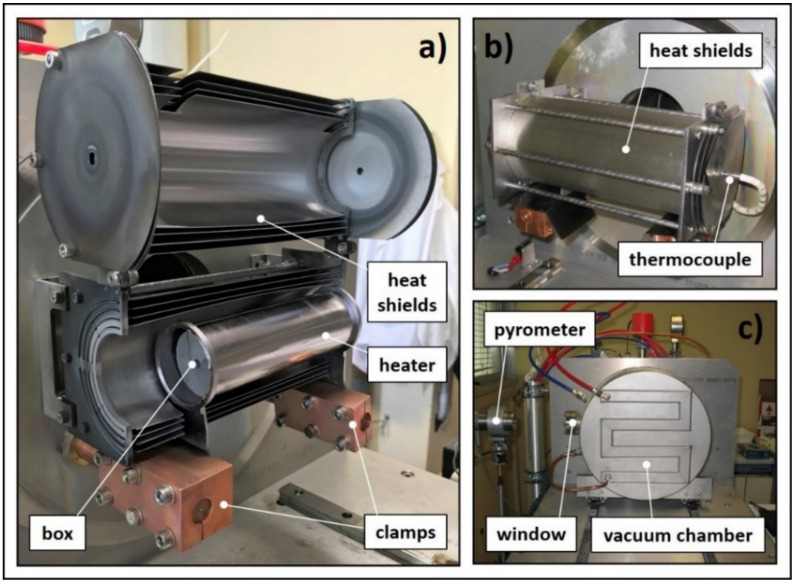
The experimental apparatus used to determine the temperature limit of target materials; (**a**) picture of the opened hot zone; (**b**) picture of the closed hot zone; (**c**) picture of the water-cooled vacuum chamber and of the temperature measurement setup.

Data related to T_LIMIT_ were collected by means of the high temperature furnace represented in Figure 5. The hot zone is composed of a tantalum tubular heater (internal diameter and thickness equal to 50 and 0.25 mm, respectively) connected to the power supply (I_MAX_ = 1300 A, V_MAX_ = 10 V) by means of two water-cooled copper clamps. In this way, it is possible to heat by Joule effect the cylindrical graphite box containing the samples (see Figure 5). Tantalum and molybdenum heat shields surround the heater, contributing to keeping the box at extremely high temperatures and to improving the homogeneity of the thermal field. The hot zone is closed inside a water-cooled vacuum chamber equipped with a boro-silicate glass window; through it an infrared pyrometer allows for temperature measurements directly inside the box, making use of a series of holes passing through the shields and the box itself. At the other side of the heat shields, another system of holes is used for the positioning of a type C thermocouple on the external cap of the box. Additionally, for this apparatus, all data are collected automatically by a dedicated PLC.

## 4. Mechanical Characterization and Related Experimental Procedures

As discussed in the previous paragraphs, the target design can be validated from the structural point of view only if the maximum stress is well below σ_LIMIT_. Taking into consideration steady-state conditions and the hypothesis of isotropic, linear elastic material, the transition from the temperature field {T} to the stress field {σ} can be made through the thermoelastic material properties E (Young’s modulus), ν (Poisson’s ratio) and α (coefficient of thermal expansion). This step is clearly illustrated in Figure 3. For standard commercial materials, these properties are usually well known, whereas for homemade materials a specific strategy for their determination is required. Different solutions are proposed in the literature [19,20,21], but when data sets are requested for radioactive materials at high temperature ranges, E, ν and α values are extremely difficult to obtain.

In the following paragraphs, the main steps adopted to estimate σ_LIMIT_ are presented. For both SiC SA and SP, the material properties E, ν and α are known parameters, and the calculation of the stress limit σ_LIMIT_ can be directly performed. On the other side, if E, ν and α are not available (this is usually the case of homemade materials for target production), the Virtual Thermoelastic Parameters approach can be adopted. Both procedures are presented in the following.

### 4.1. Stress Limit Estimated by Means of Real Thermoelastic Parameters (RTPs)

The estimation of the stress limit is based on a statistical approach and consequently 38 disc-shaped test specimens were prepared for SiC SA, and 28 for SiC SP. The diameter was fixed at 40 mm, whereas the thickness ranged from 0.5 to 1.5 mm because of evident difficulties in the manufacturing procedure: the extremely high hardness of SiC made cut operations complicated and difficult to control in terms of reproducibility.

**Figure 6:**
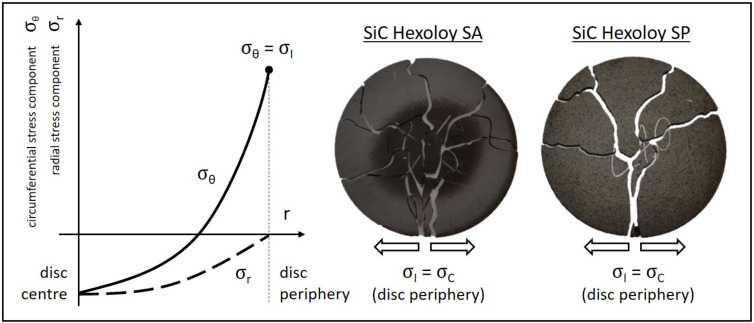
Stress components characterizing the disc-shaped test specimen and picture of two reconstructed discs evidencing the typical crack paths and crack path branches characterizing SiC SA and SiC SP samples.

Every disc was accurately positioned and centered on the experimental apparatus illustrated in Figure 4. The induced temperature field {T} proved to be axial-symmetric and characterized by strong radial temperature gradients, with decreasing temperature values from the center to the periphery. For each test, the heating current was gradually and slowly increased, augmenting at the same time thermal gradients. These are directly related to specific radial distributions of the stress components σ_θ_ and σ_r_ (σ_z_ is always negligible because of evident plane stress conditions) [22,23], as shown in Figure 6. The compressive stresses (negative values for both σ_θ_ and σ_r_) at the center of the disc do not constitute a serious problem for the structural integrity of the sample. The real danger is the peak value of the circumferential stress component σ_θ_ at the periphery of the disc. In this region, σ_θ_ reaches its maximum positive (tensile stress) value and coincides with the first principal stress σ_I_, which is, in general, used to recognize the maximum tensile stress induced by loading conditions. While increasing the heating current, the maximum value of σ_I_ (at the periphery of the disc) augments too, and when it reaches the critical value σ_C_ the test specimen fails in a brittle fashion, with the typical crack path illustrated in Figure 6. In this phase of the test, the optical pyrometer used to monitor the disc temperature detects a strong discontinuity. Indeed, the test specimen is broken into a lot of different fragments, allowing in this way the pyrometer to directly point the heater, which is much hotter. At this point, the test is stopped and the critical heating current value I_C_ can be easily obtained from recorded data.

Once the I_C_ value for the specific sample is collected, the experimental activity can be considered completed and it is possible to go on with the calculation of the critical temperature field {T_C_} making use of the electrical–thermal finite element model mentioned in Section 3.2. In this phase, the disc thermal properties ε and k adopted for calculations are those estimated according to the thermal characterization described above. In addition, the model geometry includes all the specific information regarding the sample thickness and positioning with respect to the heater.

The next step is the calculation of the critical stress field {σ_C_} according to the scheme reported in Figure 3 and making use of the Real Thermoelastic Parameters (RTPs) listed in Table 1. Once the solution is obtained, it is relatively easy to plot the first principal stress σ_I_ and to highlight the maximum value at the periphery of the disc. As shown in Figure 6, it corresponds to the critical stress σ_C_, which is in practice the maximum stress in the test specimen at failure.

The same procedure was adopted for all SiC SA and SP test specimens, obtaining in this way two series of critical stress values σ_Ci_. The resulting failure stress data were used to obtain the estimation of the Weibull probability distribution parameters according to the ASTM standard practice [24]. Then, the stress limit σ_LIMIT_ was calculated for both SiC SA and SP, taking as a reference a survival probability equal to 99.99%. As a final step, these values were associated with a specific temperature range, considering the average minimum temperature and the average maximum temperature of the discs under critical conditions.

### 4.2. Stress Limit Estimated by Means of Virtual Thermoelastic Parameters (VTPs)

**Figure 7:**
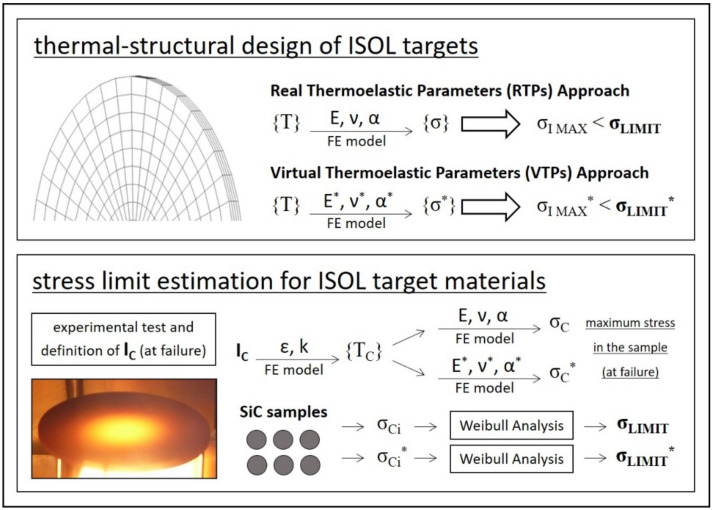
Thermal–structural design of ISOL targets making use of the RTPs and VTPs approaches.

As indicated at the beginning of the paragraph, thermoelastic material properties E, ν and α are usually unknown for most homemade ISOL materials (transition metal or actinide carbides) [26,27]. In this case, the RTPs approach cannot be used for the thermal–structural design of ISOL targets, and an alternative solution was formulated. It is clearly illustrated in Figure 7 and named as the Virtual Thermoelastic Parameters (VTPs) approach. According to it, a set of Virtual Thermoelastic Parameters E*, ν* and α* can be arbitrarily assumed. For the sake of simplicity, in this work, E*, ν* and α* were imposed equal to 102 GPa, 0.1 and 10^−6^ °C^−1^, respectively, taking as a reference the order of magnitude of the correspondent SiC material properties. This assumption is surely strong, but reasonable. Indeed, adopting the same E*, ν* and α* values for the design phase and the stress limit estimation (see Figure 7), it is formally correct to compare σ_I MAX_* and σ_LIMIT_* for the structural verification of components.

### 4.3. Fractographic Study

The ASTM standard practice [24] clearly states the importance of fractography for the estimation of Weibull Distribution Parameters. In this work, all SiC test specimens were accurately reconstructed at the end of the destructive tests described in the previous paragraphs (see Figure 6). Unfortunately, for SiC SA samples it was impossible to identify in a clear way the critical flaws. This was mainly due to the high density and microstructural homogeneity of this specific material. Moreover, SiC SA exhibits a high mechanical strength, and the high energy values released at failure led to a fine fragmentation of the samples in the proximity of the fracture origin, with the consequent impossibility to identify the critical flaws.

**Figure 8:**
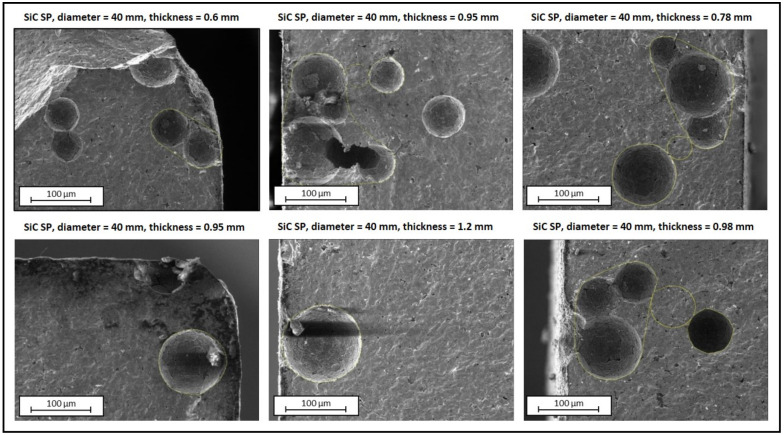
SEM images of the fracture surface in proximity of the disc periphery for six different SiC SP test specimens.

On the other side, SiC SP shows a sensibly lower mechanical strength and is characterized by discrete, non-interconnected pores, which are dispersed in a controlled manner throughout the body of the material [25]. In this case, it was possible to observe for every sample the fracture surface in the proximity of the disc periphery, with the opportunity to highlight the critical flaws originating fracture. Figure 8 presents some SEM (TESCAN, model VEGA 3xmh) images taken on the fracture surface in the proximity of the disc periphery for six different SiC SP samples. Pores are evident and their size confirms the typical values indicated in [25].

All data related to the statistical Weibull analysis presented in the next paragraph assume that all sample failures originate from the same flaw population (that is, a single failure mode).

## 5. Results and Discussion

Data for ε, k, T_LIMIT_ and σ_LIMIT_ are reported in the following for both SiC grades. These values were all obtained in a high-vacuum and high-temperature environment in order to very closely reproduce the real target material working conditions.

### 5.1. Thermal Characterization Results

Emissivity is a material surface property affected by numerous factors, such as temperature, wavelength, direction, atmosphere conditions, oxidation level, material grain size, porosity and surface roughness.

As previously mentioned, in this work the diffuse gray surface hypothesis [14] is assumed (wavelength and direction dependencies are neglected). In particular, temperature-dependent spectral normal emissivity (ε_λ,n_, λ = 1.05 μm) values were directly collected and then approximated as total hemispherical emissivity (ε) data. All emissivity values were measured in a high-vacuum and high-temperature environment with the aim to reproduce as close as possible the SPES target working conditions. The pressure inside the vacuum chamber was kept at approximately 10^−6^ mbar, substantially reducing the risk of oxidation issues. As previously discussed, the two materials studied in this work are the Hexoloy^®^ silicon carbides SA and SP, whose main characteristics are reported in Table 2. Grain size, density and pore size values were obtained directly from the manufacturer’s datasheets [25], whereas surface roughness R_a_ for both SA and SP SiC grades was directly measured at LNL (mechanical stylus method). It is important to underline that all sample discs used for thermo-mechanical characterization were cut and surface finished with the same approach adopted for the target discs that will be used for RIB production at the SPES facility. This is a very important aspect, since emissivity is a material surface property deeply influenced by surface roughness and crucial for radiative heat transfer of targets at high temperatures. SiC grades SA and SP are quite similar in grain size and density. Nevertheless, differently from the SA grade, SiC SP presents some pores that are clearly visible even on the surface. They have a relevant contribution to surface roughness, which is clearly higher for SiC SP with respect to SiC SA (see Table 2).

**Figure 9:**
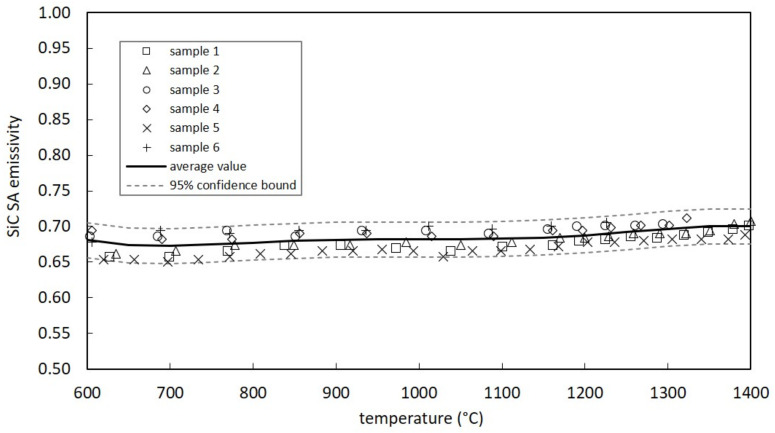
Measured emissivity values for SiC SA samples.

**Figure 10:**
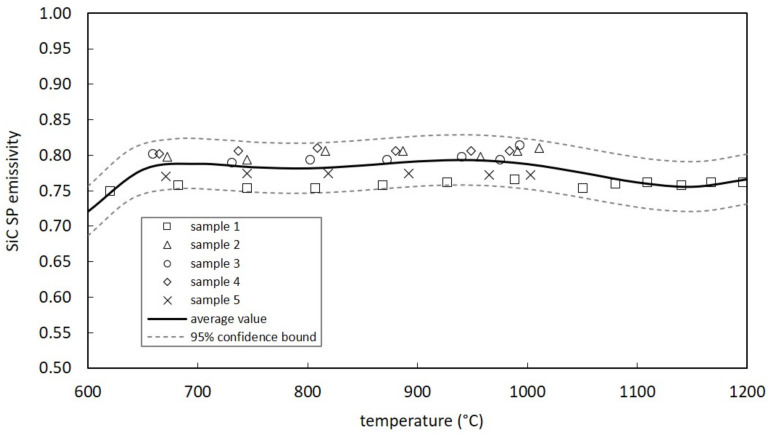
Measured emissivity values for SiC SP samples.

Figure 9 and Figure 10 report the total hemispherical emissivity (ε) data for SiC SA (six samples) and SP (five samples), respectively. With the aim to estimate the average emissivity as a function of temperature for both SiC grades, a one-variable polynomial regression model was adopted [28] using a polynomial of degree 6. Both figures show the average value, calculated according to the best polynomial regression model, with the corresponding 95% confidence intervals, derived by means of the root mean squared error.

SiC SP emissivity values (see Figure 10) are between 0.75 and 0.81, presenting evident similarities with data reported in [29] (for the lower pressure level) and in [14]. On the other side, SiC SA is characterized by a sensibly lower emissivity (see Figure 9), between 0.65 and 0.71. The lower surface roughness with respect to SiC SP and the absence of pores are two relevant aspects, and surely contribute to explain the emissivity difference between SA and SP grades. The decrease in emissivity with surface finishing is a well-known behavior, as shown in [14,30].

**Figure 11:**
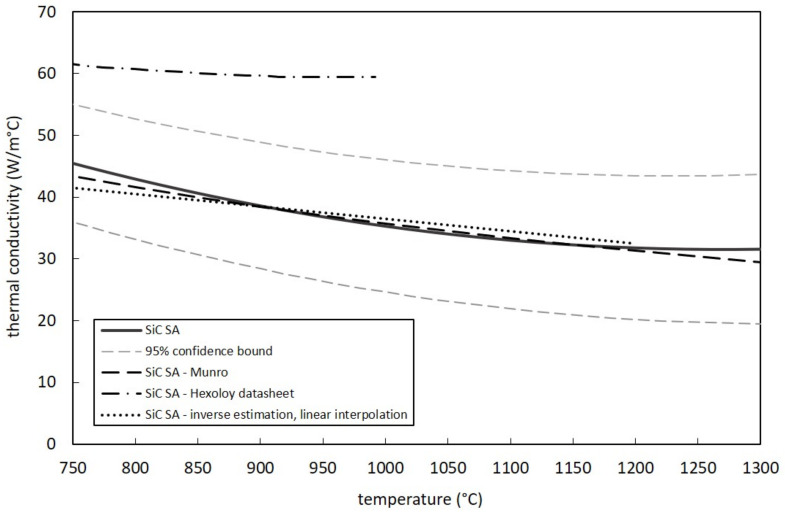
Measured thermal conductivity values for SiC SA samples and comparison with data obtained by Munro [19], Hexoloy^®^ datasheets [25] and the inverse estimation (with linear interpolation) by Manzolaro et al. [15].

**Figure 12:**
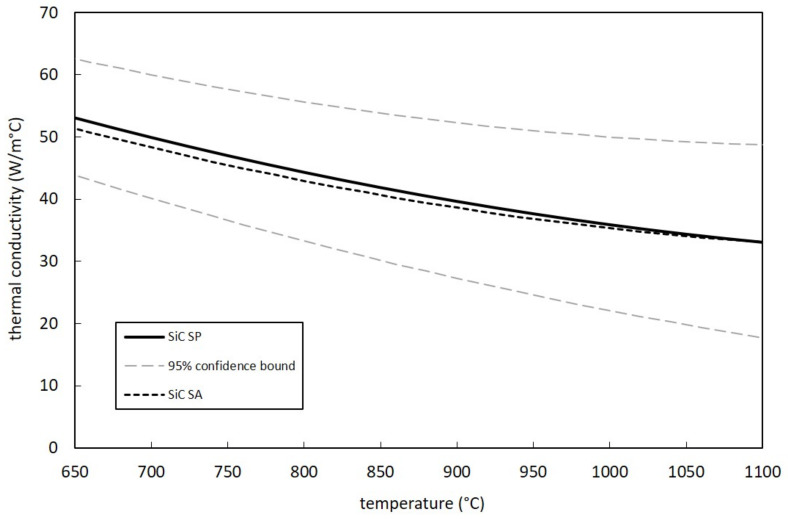
Measured thermal conductivity values for SiC SP samples and comparison with SiC SA data.

Figure 11 and Figure 12 report the thermal conductivity curves for both SiC SA and SP. They were defined by means of Equation (1), taking into consideration the parameters C_0_, C_1_ and C_2_ calculated according to the method proposed in Section 3.2 (see Table 3). In particular, Figure 11 shows the SiC SA thermal conductivity curve with the related 95% confidence bounds. The values estimated in this work are compared with data obtained by Munro [19], Hexoloy datasheets [25] and the linear estimation proposed by Manzolaro et al. [15]. All aforementioned references present a very good agreement with the proposed SiC SA thermal conductivity curve, with the only exception of Hexoloy^®^ datasheets, which clearly overestimate thermal conductivity values up to 1000 °C. All curves were plotted for temperatures between 750 and 1300 °C since all temperature measurements used for thermal conductivity calculation were observed within this range. The SiC SP curve is clearly shown in Figure 12 together with 95% confidence bounds. Additionally, in this case the temperature range was defined taking into consideration the temperature measurements performed on the sample discs. For the sake of comparison, it is possible to also appreciate in the same figure the SiC SA reference curve. Thermal conductivity values for the two SiC grades are practically identical, with differences that tend to zero at the highest temperatures. The similarities in terms of grain size and density evidenced in Table 2 surely help to explain the above mentioned coincidence of SA and SP curves.

**Figure 13:**
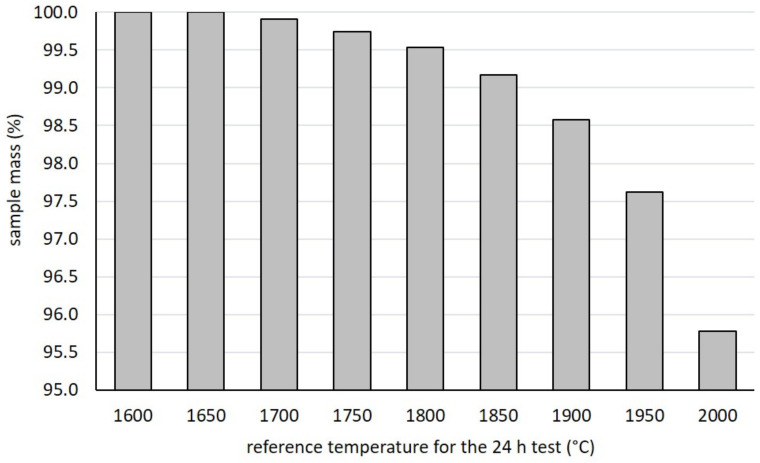
Sample mass (%) monitored at the end of every specific test (24 h at constant temperature).

As discussed in the previous paragraphs, the temperature limit (T_LIMIT_) for SiC was estimated as the highest temperature level for which the observed mass loss stays below 0.5%. Tests were performed by keeping a small SiC SA sample disc (diameter and thickness equal to 13 and 1 mm, respectively) at a constant reference temperature for 24 h and measuring its weight before and after every single test. The reference temperature for every 24 h test ranged between 1600 °C and 2000 °C, with steps of 50 °C. Tests were performed consecutively, allowing the estimation of the cumulative mass loss at the end of each one. Only SiC SA was studied, being the SP grade characterized by the same composition and grain size. Results are reported in Figure 13, showing how mass loss stays below 0.5% up to 1800 °C. For higher temperature levels, the sample mass starts to decrease significantly, with a cumulative 4.2% mass loss at 2000 °C. At the end of the tests, in light of the above considerations, T_LIMIT_ was fixed at 1800 °C.

### 5.2. Mechanical Characterization Results

**Figure 14:**
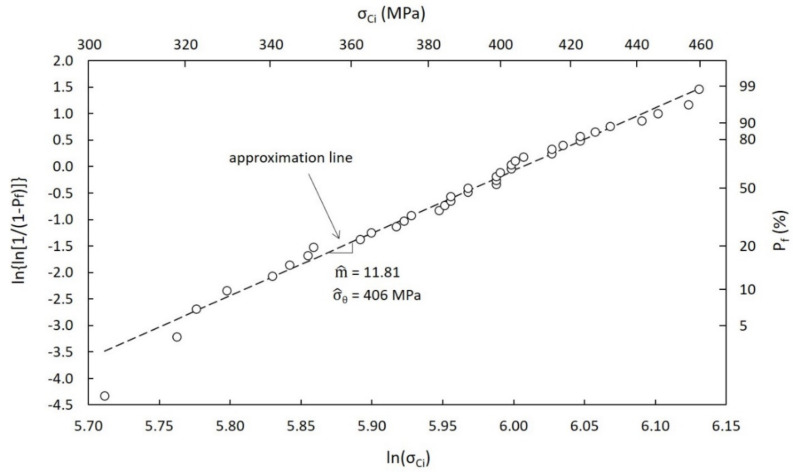
Weibull plot of strength data for SiC SA (RTPs approach).

**Figure 15:**
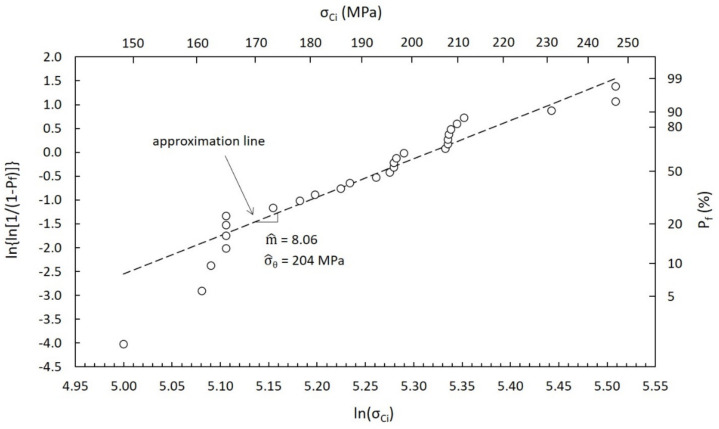
Weibull plot of strength data for SiC SP (RTPs approach).

In this paragraph, the main calculation steps followed for the definition of σ_LIMIT_ are reported. In particular, Figure 14 and Figure 15 clearly show the most important details of the statistical analyses performed for the estimation of the Weibull distribution parameters according to the ASTM standard practice [24], with P_f_ being the probability of failure and σ_Ci_ the critical stress value for the i-th sample. All input data are listed in Table 4 and Table 5 for SiC SA and SiC SP, respectively.

The estimates of the Weibull modulus $\hat{m}$ and of the Weibull characteristic strength $\hat{\sigma }$_θ_ are clearly reported in Table 6, together with their 90% confidence bounds [24]. Both SiC grades and both calculation approaches (RTPs and VTPs) were taken into consideration.

At this point, the stress limit σ_LIMIT_ was calculated for all different cases (see Table 6), taking as a reference a survival probability P_s_ equal to 99.99% (P_f_ = 1 − P_s_ = 0.01%) and the following mathematical equation (two-parameter Weibull distribution equation):

(4) $$P_{f}=1-\exp\left[-{\left(\frac{\sigma }{{\hat{\sigma }}_{\theta }}\right)}^{\hat{m}}\right]$$

As discussed in the previous paragraphs, all parameters reported in Table 6 were associated with a specific temperature range. It is easy to notice the sensibly lower strength of SiC SP with respect to SiC SA, as clearly confirmed by technical datasheets [25].

**Figure 16:**
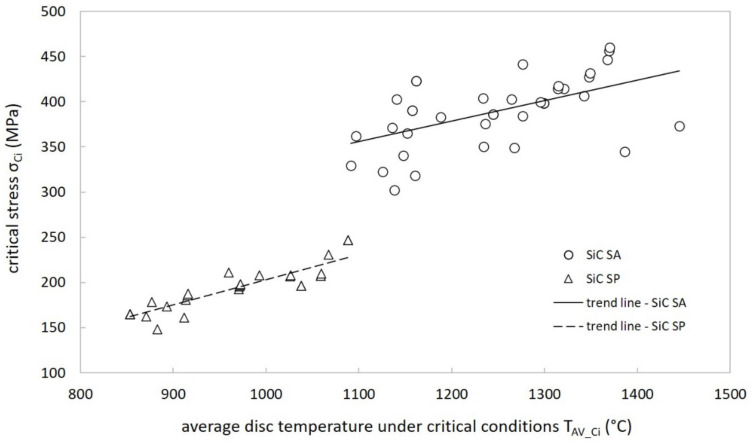
Critical stress versus temperature plot (RTPs approach) for both SiC SA and SP (T_AV_Ci_ is calculated as the arithmetic average of T_max_ and T_min_ values reported in Table 4 and Table 5).

At this point, the target designer can count on a precise stress threshold (σ_LIMIT_) to be taken as a reference for the structural verification process. The only problem could be the specified temperature range, which does not cover the typical target operation temperatures. Indeed, most of the time these are sensibly higher, very close to T_LIMIT_. Nevertheless, it is possible to observe that critical stress values σ_Ci_ reported in Table 4 and Table 5 show positive increments with increasing temperatures (see Figure 16). For this reason, all σ_LIMIT_ values reported in Table 6 should also be considered reliable for temperatures between the upper limit of the indicated temperature range and T_LIMIT_.

## 6. Conclusions

In the framework of the research and development of high-power targets for nuclear applications, the thermal and mechanical characterization of silicon carbides was carried out by means of a method based on both experimental measurements and numerical simulations. Two materials characterized by different porosities were studied in order to highlight the differences between two of the most performant materials found on the market. To obtain experimental data, two high-vacuum homemade devices were used. An extensive numerical and simulation activity was then carried out to obtain thermal and mechanical parameters. In this frame, a novel approach for the structural verification based on Virtual Thermoelastic Parameters was proposed and applied to the two types of SiC. To shed more light on the different mechanical behavior of the two materials, a fractographic study by means of SEM was also performed.

The results highlighted some peculiarities of the less porous SiC SA and the more porous SiC SP. Despite their similar microstructural properties (composition, grain size and density), SiC SP showed a higher emissivity, most probably due to a higher surface roughness. However, the thermal conductivity of the two materials did not show any significant difference in the analyzed thermal conditions. On the other hand, the mechanical characterization results showed that SiC SA has a higher stress limit due to the absence of macroscopic defects (pores) which were instead found in SiC SP. The availability of a large set of mechanical data in a defined temperature range (up to 1500 °C in the case of SiC SA) allows for the definition of parameters for the on-line operation of the high-power targets that errs on the side of safety.

All data collected at LNL for the Hexoloy^®^ silicon carbides SA and SP will be crucial for both the design phase and the commissioning of the SiC production targets that will be operated in the context of the SPES facility. As discussed in the previous paragraphs, SiC will be gradually substituted by other brand new homemade materials, with the aim to produce neutron-rich nuclei in a wide mass range (uranium carbide is surely the best candidate). Of course, the approach presented in this work for the thermal and mechanical characterizations will be extended to these new high-performance materials, allowing for a specific and well defined design procedure for the new high-performance targets to be used in the next generation ISOL facilities.

## Figures and Tables

**Table 1:** Real Thermoelastic Parameters (RTPs) for the Hexoloy^®^ silicon carbides SA [19] and SP [25].

Property—SiC SA	Units	20 °C	500 °C	1000 °C	1200 °C	1400 °C	1500 °C
Young’s modulus **E**	GPa	415	404	392	387	383	380
Poisson’s ratio **ν**	/	0.160	0.159	0.157	0.157	0.156	0.156
Thermal expansion **α**	10^−6^ °C^−1^	1.1	4.4	5.0	5.2	5.4	5.5
**Property—SiC SP**	**Units**	**20 °C**	**500 °C**	**1000 °C**	**1200 °C**	**1400 °C**	**1500 °C**
Young’s modulus **E**	GPa	400	389 *	378 *	373 *	369 *	366 *
Poisson’s ratio **ν**	/	0.140 **	0.140 **	0.140 **	0.140 **	0.140 **	0.140 **
Thermal expansion **α**	10^−6^ °C^−^^1^	1.1 ***	4.4 ***	5.0 ***	5.2 ***	5.4 ***	5.5 ***

* Young’s modulus value for SiC SP available only at Room Temperature; table completed assuming a constant value for the ratio E_SA_/E_SP_. ** SiC SP Poisson’s ratio with unspecified reference temperature; constant value (ν = 0.140) assumed for the whole temperature range. *** SiC SP coefficient of thermal expansion not available for the desired temperature range; as an approximation SiC SA values were taken as a reference.

**Table 2:** Grain size, density, pore size and surface roughness values for the Hexoloy^®^ silicon carbides SA and SP.

PROPERTY	UNITS	TYPICAL VALUE—SiC SA	TYPICAL VALUE—SiC SP
grain size	µm	4–10	4–10
density	g/cm^3^	3.10	3.04
pore size	µm	no pores	50
surface roughness R_a_	µm	0.54	1.48

**Table 3:** C_0_, C_1_ and C_2_ parameters (see Equations (1) and (2)) for the Hexoloy^®^ silicon carbides SA and SP.

MATERIAL	C_0_ (W/m°C)	C_1_ (W/m°C^2^)	C_2_ (W/m°C^3^)
SiC SA	114.527	−0.1306	0.0000514
SiC SP	115.036	−0.1254	0.0000463

**Table 4:** SiC SA input data for Weibull statistical analysis.

Sample Number	Diameter (mm)	Thickness (mm)	Volume (mm^3^)	I_c_ (A)	Tmax (°C)	Tmin (°C)	σ_c_ (MPa) (RTPs Approach)	σ_c_ (MPa) (VTPs Approach)
1	40	1.00	1257	215	1345	931	302	12.51
2	40	1.00	1257	220	1377	944	318	13.15
3	40	0.80	1005	210	1348	904	322	13.35
4	40	0.50	628	210	1322	861	330	13.54
5	40	0.80	1005	215	1379	916	340	14.05
6	30	1.00	707	250	1574	1198	344	14.15
7	40	1.45	1822	250	1491	1043	349	14.85
8	30	0.49	346	210	1432	1037	350	15.05
9	40	0.50	628	200	1348	846	362	15.06
10	40	0.50	628	240	1414	889	365	15.06
11	40	0.60	754	210	1389	883	371	15.26
12	30	1.00	707	265	1650	1240	373	15.34
13	40	1.00	1257	255	1492	980	375	15.34
14	40	0.60	754	235	1443	933	383	15.46
15	40	1.20	1508	250	1522	1031	384	15.66
16	40	1.00	1257	240	1495	994	386	15.66
17	40	1.00	1257	240	1495	994	386	15.74
18	40	0.60	754	215	1420	895	391	15.85
19	40	0.60	754	215	1420	895	391	15.85
20	30	0.50	353	225	1516	1083	398	15.85
21	30	0.50	353	225	1516	1083	399	16.03
22	30	0.50	353	225	1516	1083	399	16.09
23	40	1.20	1508	255	1548	1042	399	16.09
24	40	1.00	1257	245	1523	1006	403	16.20
25	40	0.50	628	210	1412	869	403	16.26
26	40	0.60	754	265	1514	954	404	16.45
27	40	1.45	1822	270	1595	1090	406	16.45
28	30	0.50	353	230	1544	1097	414	16.59
29	40	1.20	1508	260	1574	1054	414	16.62
30	40	1.18	1483	260	1577	1053	418	16.73
31	40	0.50	628	215	1443	881	423	17.06
32	40	0.50	628	215	1443	881	423	17.25
33	40	1.30	1634	270	1613	1082	427	17.40
34	40	1.27	1596	270	1617	1080	432	17.40
35	40	0.80	1005	265	1572	981	442	17.85
36	40	1.26	1583	275	1644	1091	447	17.86
37	40	1.20	1508	275	1652	1087	456	18.25
38	40	1.18	1483	275	1655	1086	460	18.39

**Table 5:** SiC SP input data for Weibull statistical analysis.

Sample Number	Diameter (mm)	Thickness (mm)	Volume (mm^3^)	I_c_ (A)	Tmax (°C)	Tmin (°C)	σ_c_ (MPa) (RTPs Approach)	σ_c_ (MPa) (VTPs Approach)
1	40	0.78	980	180	1011	755	148	6.95
2	40	0.97	1219	180	1047	776	161	7.46
3	40	0.59	741	175	1013	728	162	7.65
4	40	0.50	628	170	1001	706	165	7.83
5	40	0.50	628	170	1001	706	165	7.83
6	40	0.50	628	170	1001	706	165	7.83
7	40	0.50	628	170	1001	706	165	7.83
8	40	0.60	754	180	1044	742	173	8.10
9	40	0.50	628	175	1034	720	178	8.39
10	40	0.63	792	185	1069	758	181	8.40
11	40	0.60	754	185	1076	756	186	8.63
12	40	0.59	741	185	1078	755	187	8.71
13	40	0.80	1005	200	1131	810	193	8.80
14	40	0.78	980	200	1134	809	195	8.81
15	40	1.20	1508	220	1195	881	196	8.81
16	40	1.20	1508	220	1195	881	196	8.93
17	40	0.77	968	200	1136	808	197	8.99
18	40	0.76	955	200	1138	807	198	9.06
19	40	0.98	1232	215	1193	858	207	9.29
20	40	1.20	1508	225	1223	894	208	9.29
21	40	1.20	1508	225	1223	894	208	9.33
22	40	0.78	980	205	1164	821	208	9.38
23	40	0.97	1219	215	1195	858	208	9.38
24	40	1.18	1483	225	1226	893	209	9.44
25	40	0.60	754	195	1138	781	211	9.70
26	40	0.98	1232	225	1251	883	231	10.34
27	40	0.95	1194	230	1284	894	247	11.01
28	40	0.95	1194	230	1284	894	247	11.01

**Table 6:** Weibull distribution parameters (with 90% confidence bounds) and calculated stress limit values.

Specific Case	$\hat{m}$ (/) (Lower Bound)	$\hat{m}$ (/) (Average)	$\hat{m}$ (/) (Upper Bound)	$\hat{\sigma }$_θ_ (MPa) (Lower Bound)	$\hat{\sigma }$_θ_ (MPa) (Average)	$\hat{\sigma }$_θ_ (MPa) (Upper Bound)	σ_LIMIT_ (MPa)	Temperature Range (°C)
SiC SA (RTPs appr.)	9.2073	11.8130	14.1473	396.10	406.05	416.35	190.00	1000 ÷ 1496
SiC SA (VTPs appr.)	10.3008	13.2159	15.8274	16.09	16.45	16.82	8.00	1000 ÷ 1496
SiC SP (RTPs appr.)	5.9651	8.0589	9.8882	195.13	203.72	212.81	65.00	803 ÷ 1124
SiC SP (VTPs appr.)	6.8004	9.1873	11.2728	8.93	9.27	9.63	3.50	803 ÷ 1124

## Data Availability

Data sharing is not applicable to this article.
